# Pelargonidin-*3*-*O*-Glucoside Encapsulated Pectin-Chitosan-Nanoliposomes Recovers Palmitic Acid-Induced Hepatocytes Injury

**DOI:** 10.3390/antiox11040623

**Published:** 2022-03-24

**Authors:** Naymul Karim, Mohammad Rezaul Islam Shishir, Yuting Li, Ould Yahia Zineb, Jianling Mo, Jitbanjong Tangpong, Wei Chen

**Affiliations:** 1 Department of Traditional Chinese Medicine, School of Medicine, Sir Run Run Shaw Hospital, Zhejiang University, Hangzhou 310016, China; naiemph@zju.edu.cn (N.K.);; 2 Department of Food Science and Nutrition, Zhejiang University, Hangzhou 310058, China; rezaul@zju.edu.cn (M.R.I.S.); 11813035@zju.edu.cn (Y.L.);; 3 Biomedical Sciences, School of Allied Health Sciences, Walailak University, Nakhon Si Thammarat 80161, Thailand; rjitbanj@wu.ac.th; 4 Ningbo Research Institute, Zhejiang University, Ningbo 315100, China

**Keywords:** liposomes, chitosan, pectin, pelargonidin-*3*-*O*-glucoside, palmitic acid, lipotoxicity

## Abstract

Pelargonidin-*3*-*O*-glucoside (Pg) is a well-known anthocyanin derivative possessing potential biological activity. Nonetheless, the bioactivity of Pg is limited due to instability in the physiological environment. Functionalized nanoliposomes using chitosan and/or pectin coating is an excellent carrier system for nanoencapsulation of food bioactive compounds such as Pg. Therefore, this study aimed to investigate the protective effect of Pg-loaded pectin–chitosan coated nanoliposomes against palmitic acid (PA)-induced hepatocytes injury in L02 cells. Firstly, Pg-loaded pectin–chitosan coated nanoliposomes were characterized using the DLS, HPLC, TEM, and cellular uptake study in L02 cells. Thereafter, we assayed the protective effect against PA-induced lipotoxicity, ROS and O_2_^•−^ generation, mitochondrial dysfunction (MMP), and GSH depletion. Results showed that Pg-loaded nanoliposomes significantly reduced the PA-induced L02 cells toxicity via suppressing ROS production, O_2_^•−^ generation, MMP collapse, and GSH reduction, whereas the free-Pg samples were not effective. On the contrary, the chitosan and/or pectin coated nanoliposomes showed higher results compared to coating-free nanoliposomes. Altogether, the results of our study ensured that Pg-loaded pectin–chitosan coated nanoliposomes was capable of reducing PA-induced hepatocytes injury. Thus, pectin–chitosan coated nanoliposomes can be useful for hepatocellular delivery of hydrophilic compounds with greater biological activity.

## 1. Introduction

Pelargonidin-*3*-*O*-glucoside (Pg) is a well-known anthocyanin derivative, which is rich in berry fruits, especially in strawberries. Pg consists of pelargonidin with a *β*-D-glucosyl residue that is attached at the 3′-hydroxy position of C-ring. It also carries one hydroxyl group at the 4′-position of B-ring [1]. Pg processes a wide range of biological activity including antioxidant [1], anti-inflammatory [2], anti-hyperglycemic [3], antiobesity [4], antidiabetic [5], neuroprotective [6], and anticancer activity [7]. Several physicochemical or physiological instabilities, such as physiologic pH, temperature (37 °C), oxygen, salt, enzymes, etc., limit the therapeutic effect of Pg [8]. Liposomal encapsulation (liposomes is self-assembled lipid vesicular system) technique is one of the better ways to improve the therapeutic activity of encapsulated-compounds [9]. Liposomes can successfully protect the environmentally sensitive bioactive compounds via encapsulating them inside the carrier system. In addition, the surface decoration of liposomes by the biopolymers (such as pectin, chitosan, alginate, and others) can further improve the physicochemical stability and controlled release properties [10]. For example, indocyanine green (ICG) is a promising candidate for the photodynamic therapy of topical melanoma, but unstable and hardly permeable through skin. Encapsulation of ICG to chitosan-coated liposomes reduced the ICG degradation and enhanced the skin permeability [11]. Surface decoration of curcumin-loaded liposomes by chitosan further increased the stability and bioavailability compared to curcumin-loaded uncoated-liposomes [12]. Succinyl-chitosan coating on to liposomes improved the gastric stability of liposomal vesicles and increased the co-delivery of quercetin and resveratrol under the simulated intestinal fluid [13]. On the other hand, pectin coating is also a promising way for improving the stability and mucoadhesive property of liposomes, which can improve the colonic delivery of liposomes [14]. Pectin-coated liposomes is also effective for the delivery of amoxicillin to combat the *Helicobacter pylori* infection via specific targeting against BabA/LPS of *H. pylori* and inhibiting bacterial adhesion of *H. pylori* to human host cells [15]. Eudragit-coated liposomes improved the gastrointestinal stability and antioxidant activity of quercetin [16]. Several researchers used the dual or multiple coating to further improve the physicochemical stability, oral delivery, controlled release property, and biological activity of liposomal systems, such as chitosan–xanthan gum coated liposomes [17], alginate-chitosan coated liposomes [18], pectin-whey protein coated liposomes [19], etc.

Palmitic acid (PA) is a well-known abundant free fatty acid, which can be found in the human body and diet. PA is responsible for cell dysfunction and death at an excess level called “lipotoxicity”. PA-induced lipotoxicity is involved in the pathogenesis of metabolic diseases such as insulin resistance, obesity, type 2 diabetes, atherosclerosis, coronary heart disease, and non-alcoholic fatty liver disease [20,21,22]. A myriad of studies revealed that elevated levels of reactive oxygen species (ROS) or oxidative stress are the pathological results of PA-induced lipotoxicity [4,23]. A study reported that PA treatment induced the cellular apoptosis in Chang liver cells via increasing mitochondrial alterations, which was caused by oxidative stress [24]. In contrast, another study revealed that the excessive production of ROS were found in PA-induced hepatoma cells, indicating that ROS are the prime mediator of cellular lipotoxicity [25]. Naturally-obtained antioxidant compounds lessen the lipotoxicity in hepatocytes via reducing oxidative stress [26]. Thus, effective delivery of antioxidant compounds could suppress the hepatocytes’ injury via exerting antioxidant activity.

In our previous study, we observed that pectin and/or chitosan coating not only improved the physicochemical stability of Pg-loaded nanoliposomes, but also ensured the controlled release properties compared to uncoated-nanoliposomes. Our current study is the connecting work of our previously published article [8]. Here, we have investigated the protective effect of Pg-loaded pectin–chitosan coated nanoliposomal system against palmitic acid (PA)-induced hepatocytes injury in normal human hepatocytes (L02 cells). We hypothesized that Pg-loaded pectin–chitosan coated nanoliposomal system could exert better outcomes in the amelioration of PA-induced hepatocytes injury than that of free-Pg or Pg-loaded uncoated-nanoliposomes. The characterization of nanoliposomal particles was performed using dynamic light scattering (DLS), HPLC, and transmission electron microscopy (TEM). Afterward, we evaluated the cytotoxicity, cytoprotectivity, hepatocellular uptake, and oxidative stress (e.g., ROS, O_2_^•−^, MMP, and GSH)-related studies using primary hepatocytes (L02 cells).

## 2. Materials and Methods

### 2.1. Chemicals and Reagents

Pelargonidin-*3*-*O*-glucoside (Pg, purity 97.26%) was isolated from strawberry fruits in our laboratory and stored at −80 °C until used [1]. The soybean lecithin and cholesterol for preparing nanoliposomes were purchased from Sinopharm Chemical Reagent Co. Ltd., Shanghai, China. For nanoliposomes coating, the Chitosan (Specifications: 85% [degree of deacetylation], 50 kDa [molecular weight], and S24914 [lot number]) was bought from Shanghai yuanye Bio-Technology Co. Ltd., Shanghai, China, while the Pectin from citrus peel (Specifications: ≥74.0% [galacturonic acid], fine powder [dry basis], and SLBV5461 [lot number]) was purchased from Sigma-Aldrich, St. Louis, MO, USA. The chemicals used for cellular studies, such as 3-(4,5-dimethyl-2-thiazolyl)-2,5-diphenyl-2-Htetrazolium bromide (MTT), Palmitic acid (PA), Rhodamine 123 (RH123), 2′,7′-dichlorofluorescein diacetate (DCFH-DA), Naphthalene-2,3-dicarboxyaldehyde (NDA), and Dihydroethidium (DHE), were bought from Sigma-Aldrich, St. Louis, MO, USA. Analytical grade reagents were used for all experiments.

### 2.2. Nanoliposomes Formation

The nanoliposomes (NL) were prepared according to our previously published article with some amendments [27]. In brief, soybean lecithin and cholesterol (mass ratio of 6:1) were mixed in absolute ethanol for 15 min using a vortex mixer. Then, the mixture was evaporated at 60 °C using a rotary evaporator to form the thin dried lipid film. Thereafter, lipid film was rehydrated by 0.05 M phosphate buffer saline (PBS, pH 7.4), which contained 8 mg of Pg. After that, a mechanical force was applied for 30 min via vortex mixer and then permitted to stand for another 30 min to form the visible primary micro-sized liposomes. Next, an 8 min probe sonication was applied (ultrasonic time was set 75% with full sonication power 80% of 150 W) using JY 96-II probe sonicator (Scientz Biotechnology, Ningbo, China) in order to make the invisible nanoliposomes from primary liposomes. Then, the final Pg-loaded nanoliposomes (Pg-NL) was preserved at refrigerator (4 °C) for future study.

### 2.3. Modification of Nanoliposomes Surface Using Chitosan and Pectin Coating

First, the stock solutions (1 wt%) of chitosan and pectin were prepared following our previous article [27]. Next, 0.6 wt% of chitosan was bound with nanoliposomes (at a ratio of 1:1 *v*/*v*) to form the chitosan-coated nanoliposomes (CH-Pg-NL). Then, 0.5 wt% of pectin was bound with chitosan-coated nanoliposomes (at a ratio of 1:1 *v*/*v*) to form the pectin-chitosan-coated nanoliposomes (P-CH-Pg-NL) [8]. 

### 2.4. Characterization of Different Liposomal Particles

#### 2.4.1. Dynamic Light Scattering (DLS)

After formation of all particles, the DLS method was applied using Malvern ZetasizerNano-ZS90 (Malvern Instruments Ltd., Worcestershire, UK) to measure the particle size (nm), surface charge (mV), and polydispersity index (PDI). The Malvern ZetasizerNano-ZS90 was equipped with He/Ne laser operating at a wavelength of 633 nm. In our study, the 12 mm Square Polystyrene Cuvettes (DTS0012) and Malvern Panalytical Disposable Capillary Cells (DTS1070) were used for Mean particle size and Particle surface charge, respectively. For analyzing particle size and PDI, we selected the following conditions. Materials: Liposome; Dispersant, Water; Temperature, 25.0 °C; Viscosity, 0.8872 cP; Refractive index (RI), 1.330; Equilibration time, 60 s; Scattering angle, 90°; and Measurement duration, Automatic. For analyzing Zeta potential, we selected the following conditions. Materials, Liposome; RI, 1.450; Absorption, 0.001; Dispersant, Water; Temperature, 25.0 °C; Viscosity, 0.8872 cP; Refractive index (RI), 1.330; Dielectric constant, 78.5; Model, Smoluchowski; F (Ka) value, 1.50; Equilibration time, 120 s; and Measurement duration, Automatic. Firstly, nanoliposomes samples were diluted 10 times in phosphate buffer saline to avoid the multiple scattering effects. For DLS analysis, 1 mL diluted nanoliposomal sample was put in the machine. All data were calculated as the mean value after triplicate measurements.

#### 2.4.2. Encapsulation Efficiency (EE)

The encapsulation efficiencies (EE) were determined according to a previous study with slight modifications [10]. Firstly, encapsulated-Pg was separated from non-encapsulated Pg through refrigerated centrifugation at 20,000× *g* for 60 min (TGL-16M, Shanghai Lu Xiangyi centrifuge instrument Co., Ltd., Shanghai, China). Thereafter, the supernatant was collected for the determination of free-Pg. To determine the encapsulated Pg, the precipitates of liposomal pellets were collected followed by the disruption of pellets using Triton X-100 (6% *v*/*v*). Next, the disrupted nanoliposomes and collected supernatant were filtered using a 0.22 µm filter. The absorbance of Pg was taken at 280 nm using an HPLC, which was equipped with diode array detection (Dionex ultimate 3000, ThermoFisher Scientific, Waltham, MA, USA) [1]. The EE (%) of Pg was calculated using the below equation.

(1) $$\mathrm{EE}\;\left(\%\right)=\frac{\mathrm{Encapsulated}\;\mathrm{Pg}}{\left(\mathrm{Free}\;\mathrm{Pg}+\mathrm{Encapsulated}\;\mathrm{Pg}\right)}\times \;100$$

#### 2.4.3. Transmission Electron Microscope (TEM)

A JEM-1200EX transmission electron microscope (Japanese Electronics Co., Ltd., Tokyo, Japan) was used to evaluate the morphological appearance and cargo loading of nanoliposomal samples. Briefly, fresh nanoliposomes were diluted and then transferred on a 200-mesh carbon-coated copper grid. After that, the sample on copper grid was negatively stained by one drop of 1% phosphotungstic acid solution (pH 6.5) for 1 min followed by drying at room temperature. TEM images were taken at 80 kV accelerating voltage [10].

### 2.5. Cell Culture

Normal primary human hepatocyte cells line (L02) were purchased from the Type Culture Collection of the Chinese Academy of Sciences (Shanghai, China). Afterward, L02 cells were cultured in RPMI 1640 medium (Gibco), which contained penicillin (100 units/mL), streptomycin (100 units/mL), and fetal bovine serum (10%). After that, cells were incubated in an incubator at 37 °C with 5% CO_2_.

#### 2.5.1. Cytotoxicity Study

The cytotoxicity studies were conducted by MTT assay following previously published literature [28,29]. In brief, L02 cells were seeded into a 96-well plate at a density of 5 × 10^3^ well and cultured for 24 h. Then, cells were pretreated with several concentrations of free-Pg and Pg-loaded nanoliposomes (1.6, 3.2, 4.8, 6.4, and 8 µM) for another 24 h. In our study, the doses of all nanoliposomal samples were calculated based encapsulated-Pg, whereas the encapsulated-Pg were measured once in a week by HPLC for cellular studies. Next, cells were washed two times with PBS and then incubated for 4 h with MTT reagent (0.5 mg/mL). Then, 150 μL DMSO was added to dissolve the produced-formazan precipitate, and then absorbance was measured at 490 nm using Tecan Infinite M200 microplate reader.

#### 2.5.2. Cytoprotective Study

The cytoprotective effects of free-Pg and Pg-loaded nanoliposomes were evaluated against palmitic acid (PA) induced-cytotoxicity [28,29]. Firstly, L02 cells were seeded into a 96-well plate at a density of 5 × 10^3^ well and cultured for 24 h. Then, cells were washed two times with PBS, and pre-treated with samples at two different concentrations (4 and 8 µM) for 24, 48, and 72 h. After the incubation period, PA (0.15 mM) was added to cells for another 24 h. After that, 150 μL DMSO was added to dissolve the produced-formazan precipitate, and then absorbance was measured at 490 nm using Tecan Infinite M200 microplate reader.

#### 2.5.3. Evaluation of Cellular Uptake of Liposomal Particles to L02 Cells

The cellular uptake of liposomal particles to L02 cells was conducted according to previous protocol with slight amendments [30]. Briefly, L02 cells were seeded into a 35 mm cell culture dish at a density of 2 × 10^5^ cells and incubated for 24 h. Thereafter, L02 cells were washed two times with PBS and then added Rhodamine 123 (RM, 5 μM)-loaded liposomal samples. After incubation at 37 °C for 4, 8, and 12 h, samples were removed and washed three times with PBS. Next, all cells were immediately examined under a fluorescence microscope. Results were recorded as the “mean RM fluorescence intensity”. The data were calculated as the mean of six different microscopic fields using ImageProPlus 6.0 (Media Cybernetics Inc., Rockville, MD, USA).

#### 2.5.4. Determination of Intracellular Reactive Oxygen Species (ROS) Generation

The intracellular ROS generation were evaluated following the previously described methods with slight modifications [4,31]. The intracellular ROS generation were measured using DCFH-DA (2,7-dichlorodihydrofluorescein diacetate) fluorescent dye. In short, L02 cells were seeded for 24 h into a 24 well plate at a density of 4 × 10^4^. Then, liposomal samples were added to L02 cells for 48 h. After the incubation period, PA (0.15 mM) was added to cells for another 24 h. Afterward, cells were washed two times with PBS and then stained with DCFA-DA (10 μM) at 37 °C for 30 min. Thereafter, the unbound dye was removed by rinsing the cells with PBS thrice. Then, the fluorescence intensity was observed under a fluorescence microscope, while data were calculated using Image-Pro Plus 6.0 (Media Cybernetics, Inc., Rockville, MD, USA). The data were presented as the “mean DCF fluorescence intensity” of six different microscopic fields. 

#### 2.5.5. Determination of Superoxide Anion Radical (O_2_^•−^) Generation

The intracellular O_2_^•−^ generation were evaluated following the previously described methods with slight modifications [4,31]. The intracellular O_2_^•−^ generation were measured using DHE (dihydroethidium) fluorescent dye. The treatment protocol was similar to ROS measurement, while DHE (10 μM) was added to L02 cells for 30 min at 37 °C. The data were calculated using Image-Pro Plus 6.0 (Media Cybernetics, Inc.). The data were presented as the “mean DHE fluorescence intensity” of six different microscopic fields.

#### 2.5.6. Evaluation of Cellular Mitochondrial Membrane Potential (MMP)

Cellular MMP were conducted following earlier protocol [4,28]. The liposomal treatments for MMP were similar to ROS, while cells were incubated at 37 °C for 30 min with the Rhodamine123 (RH123, 10 μg/mL). After washing three times with PBS, results were quantified and presented as “mean RH 123 fluorescence intensity”.

#### 2.5.7. Evaluation of Intracellular Glutathione (GSH) Content

The intracellular GSH content were determined according to previous literature with minor amendments [4]. The liposomal treatments for GSH were similar to ROS, while cells were incubated at 37 °C for 30 min with the naphthalene-2,3-dicarboxyaldehyde (NDA, 50 μM). After washing three times with PBS, results were quantified and presented as “mean NDA fluorescence intensity”.

### 2.6. Statistical Analysis

Data were presented as mean ± standard error mean (SEM), whereas multiple comparisons were evaluated by one-way ANOVA followed by Duncan test using SPSS (IBM SPSS, V. 17.0, New York, NY, USA). *p* < 0.05 was considered as statistically significant. In addition, all analyses were carried out in triplicates.

## 3. Results and Discussion

### 3.1. Characteristics of Pg-Loaded Nanoliposomal Carriers

**Figure 1:**
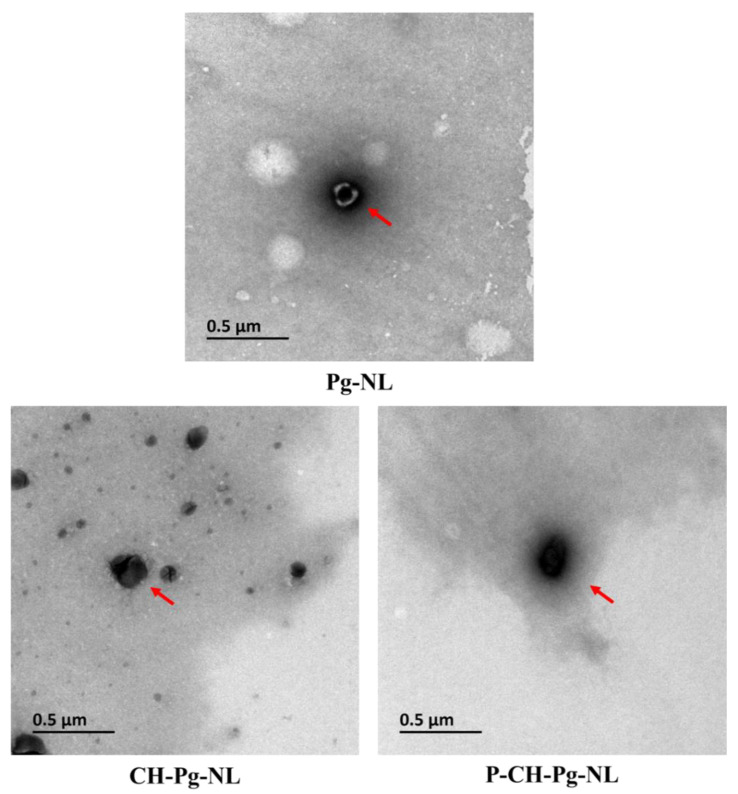
Transmission electron microscopy (TEM) analysis of Pg-loaded liposomal systems. Here, Pg-NL = Pelargonidin-*3-O*-glucoside-loaded uncoated nanoliposomes; CH-Pg-NL = Pg-NL coated by chitosan; P-CH-Pg-NL = CH-Pg-NL coated by pectin.

Table 1 and Figure 1 showed the characteristics of Pg-loaded nanoliposomal carriers. Table 1 represented the particle size, charges, polydispersity index, and encapsulation efficiency. The uncoated Pg-loaded nanoliposomes (Pg-NL) exhibited particle size ~65 nm and surface charge −30 mV. Chitosan and then pectin coating on to Pg-NL increased the particle size from 64 to 252 to 352 nm, respectively, while the surface charge was alternated from negative (−30 mV) to positive (+21) to negative (−20 mV), respectively. Both criteria, increase of particle size and alternation of particle charges, confirmed the successful coating on to Pg-NL by chitosan (CH-Pg-NL) and then by pectin (P-CH-Pg-NL) [29]. The polydispersity index (PDI) values of all Pg-loaded liposomal carriers were within the range of 0.13 to 0.24. Actually, PDI range from 0 to 0.3 indicates the homogeneous particle dispersion in the solvent, while PDI range above 0.3 indicates the heterogenic solution [32]. Thus, the developed liposomal systems were of homogeneous dispersion.

The HPLC analysis proved the well encapsulation of Pg in liposomal systems. The Pg is a hydrophilic compound, therefore, it was encapsulated into the inner aqueous core of nanoliposomes. According to HPLC analysis, the coated-nanoliposomes showed higher encapsulation efficiency (~53 and 61% for the chitosan and pectin, accordingly) than that of uncoated-nanoliposomes (Table 1). The biopolymer (such as chitosan, pectin, etc.) coating on to nanoliposomes can reduce the liposomal membrane fluidity and drug leakage, and, thereby, can increase the encapsulation efficiency [33,34,35]. 

The morphological features and drug loading in liposomal systems were confirmed by TEM (Figure 1). All nanoliposomal systems were smooth surfaces, close to spherical shape, and small unilamellar vesicles. Pg-NL had outer visible phospholipid bilayer and inside blackish color, which indicates the drug loading. However, the CH-Pg-NL showed the solid black color with invisible phospholipid bilayer and drug, which confirms the successful coating of Pg-NL by chitosan. In contrast, we found an ash-like shadow layer in P-CH-Pg-NL particle. In addition, the size of P-CH-Pg-NL was bigger than that of CH-Pg-NL, which confirms the coating of pectin on to CH-Pg-NL. These results were supported by the previously published research [10,35]. Therefore, both DLS and TEM results confirm that uncoated-nanoliposomes had smaller particle size; consequently, electrostatic chitosan coating and then pectin coating significantly increased the particle size (Table 1 and Figure 1).

### 3.2. Effect of Free-Pg and Pg-Loaded Liposomal Systems on Cell Viability

**Figure 2:**
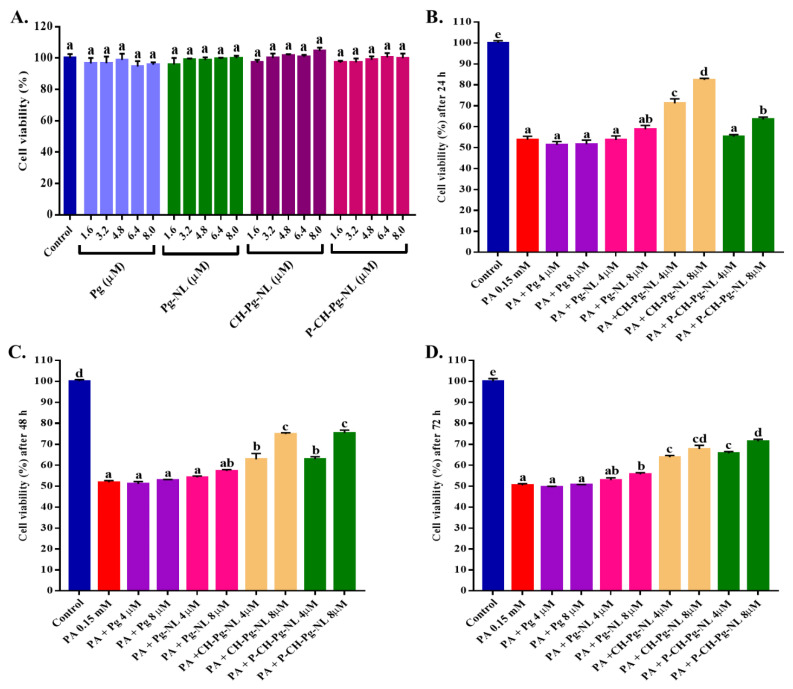
Effects of free-Pg and Pg-loaded liposomal systems on cytotoxicity and PA-induced lipotoxicity in L02 cells. (**A**) Cells were treated for 24 h with 1.6–8.0 μM of free-Pg and Pg-loaded nanoliposomes, and cell viability was evaluated by MTT assay (% of control). (**B**–**D**) Cells were treated for 24 h with 0.15 mM PA with or without free-Pg and Pg-loaded nanoliposomes for 24, 48, and 72 h, accordingly. After that, cell viability was evaluated by MTT assay (% of control). Individual alphabet represents the significant difference (*p* < 0.05) of all treatment groups. Here, PA = palmitic acid; Pg-NL = Pelargonidin-*3-O*-glucoside-loaded uncoated nanoliposomes; CH-Pg-NL = Pg-NL coated by chitosan; P-CH-Pg-NL = CH-Pg-NL coated by pectin.

Before conducting the cytoprotective study, we first checked the effect of free-Pg and Pg-loaded liposomal systems in L02 cells using MTT assay. We chose different concentrations (1.6, 3.2, 4.8, 6.4, and 8.0 µM) from each sample based on the encapsulation efficiency of liposomal systems. As shown in Figure 2A, we did not find any significant differences of cytotoxicity of the samples than that of control. During our previous study on lipophilic compound (neohesperidin)-loaded nanoliposomes, we checked the cytotoxicity of blank- and neohesperidin-loaded nanoliposomal systems. That time, we did not observe any cytotoxic effect of blank- or neohesperidin-loaded nanoliposomal systems compared to control group [36]. Therefore, our nanoliposomal systems are safe and biocompatible. Thus, we casually selected two different concentrations (4 and 8 µM) of all liposomal samples for future studies.

### 3.3. Cellular Uptake Study of Rhodamine 123 (RM)-Loaded Liposomal Systems

**Figure 3:**
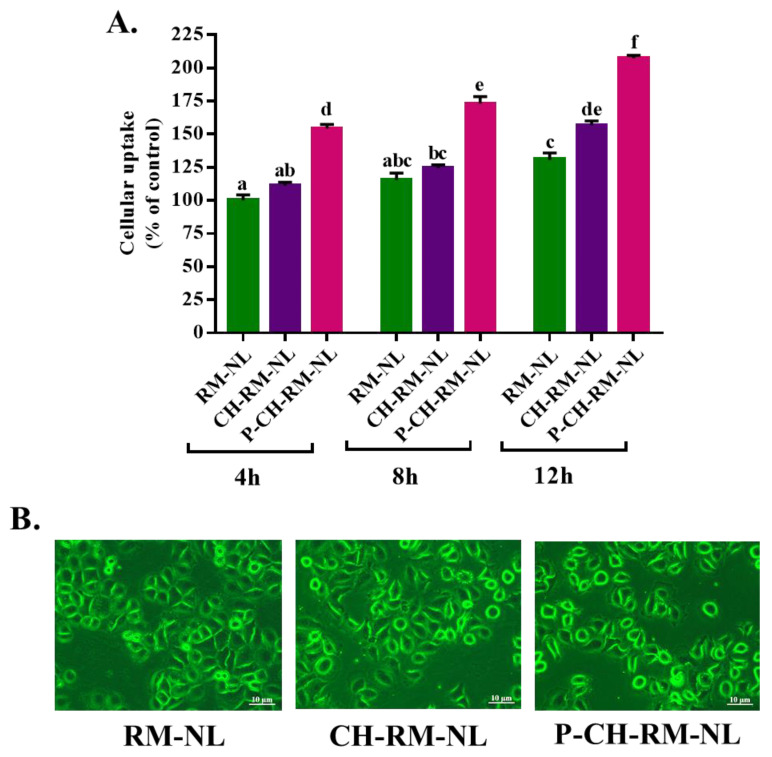
Cellular uptake of rhodamine 123 (RM, 5 μM)-loaded nanoliposomes (RM-NL, CH-RM-NL, and P-CH-RM-NL) to L02 cells. L02 cells were incubated with RM-loaded nanoliposomes for 4, 8, and 12 h, accordingly (**A**) The values of fluorescence intensity of RM-loaded nanoliposomes. Individual alphabet represents the significant difference (*p* < 0.05) of all treatment groups. (**B**) The photograph of fluorescence intensity of the RM-loaded nanoliposomes after 12 h of incubation. Here, RM-NL = rhodamine 123-loaded uncoated nanoliposomes; CH-RM-NL = RM-NL coated by chitosan; P-CH-RM-NL = CH-RM-NL coated by pectin.

To understand the cellular uptake of hydrophilic compound (Pg)-loaded liposomal systems to L02 cells, we encapsulated the hydrophilic dye Rhodamine 123 (RM) in liposomal systems. Then, we visually evaluated the cellular uptake of RM-loaded nanoliposomes to L02 cells for three consecutive times, (e.g., 4 h, 8 h and 12 h) using fluorescence microscopy. According to our results, all RM-loaded nanoliposomes were successfully uptake to L02 cells (Figure 3A). Yin et al., 2005 reported that there is no significant difference of cellular endocytosis between two particles if the particle size is below 500 nm [37]. In our study, the particle size of all liposomal systems was less than 360 nm. We also found that the cellular uptake was time-dependent (Figure 3A), and 12 h incubation time showed the highest fluorescence intensity (Figure 3B). The cellular uptake of CH-RM-NL and P-CH-RM-NL were significantly higher than that of RM-NL, which indicates that conjugation of chitosan and/or pectin enhanced the cellular uptake. This result is supported by Yin et al., 2005, who reported that biopolymers conjugation can enhance the cellular uptake to Caco2 cells [37]. Previous studies reported that two characteristics such as surface charge and increased surface hydrophilicity of biopolymers could enhance the cellular uptake of particles [38,39]. Lunov et al., 2011, reported that functional groups of biopolymers also have positive role on cellular uptake of nanoparticles. For example, the amine group (−NH_3_^+^) of chitosan and carboxylic group (−COOH) of pectin can improve the cellular uptake of nanoparticles [40]. We found that P-CH-RM-NL exhibited the highest cellular uptake, which might be due to the high methoxylated pectin (HMP, DE > 50) that exerts good cytoadhesion property [41]. Thus, HMP coating can improve the particle adhesion to cell membrane and cellular absorption. Apart from this, P-CH-RM-NL is an anionic particle because of the outer pectin coating. On the one hand, the negative charge of pectin helps the liposomal particle to interact with the positive site of cells membrane protein for endocytosis. On the other hand, P-CH-RM-NL can also be captured by cells because of the repulsive interaction with negative cells surface [42].

### 3.4. Effect of Free-Pg and Pg-Loaded Liposomal Systems against PA-Induced Lipotoxicity

Firstly, we have conducted the cytotoxicity study of different concentrations of PA (0.05 to 0.3 mM). We selected the PA concentration 0.15 mM (cell viability 53.16% compared to control) for inducing lipotoxicity. To understand the Pg release inside cells from liposomal systems, a time-dependent cytoprotective study against PA-induced lipotoxicity was conducted. According to Figure 2B, several samples’ treatment for 24 h, such as free-Pg samples (4 and 8 µM), and low concentration (4 µM) of Pg-NL and P-CH-Pg-NL, did not show cytoprotective effect compared to CH-Pg-NL samples (4 and 8 µM). In our previous study, we found that Pg-loaded uncoated nanoliposomes are unstable in the physiological temperature (37 °C), salts, pH, oxidant, and serum compared to Pg-loaded coated nanoliposomes [8]. At the 48 h (Figure 2C), both coated nanoliposomes (CH-Pg-NL and P-CH-Pg-NL) exerted similar cytoprotectivity against PA-induced L02 cells, these results were significantly (*p* < 0.05) higher compared to free-Pg and Pg-NL samples. Interestingly, pectin–chitosan coated nanoliposomes (P-CH-Pg-NL) exerted comparatively higher cytoprotective activity at 72 h than that of only chitosan-coated nanoliposomes (CH-Pg-NL) (Figure 2D), which confirms the better controlled release properties of pectin–chitosan coated nanoliposomes (P-CH-Pg-NL) over the CH-Pg-NL. From our cytoprotective study (Figure 2B–D) it is confirmed that the naked-Pg and Pg-NL samples were susceptible to physiological environment, whereas CH-Pg-NL showed higher release at 24 h. Based on MTT analysis from 24 to 72 h, P-CH-Pg-NL exerted time-dependent controlled release of Pg, whereas the release rate was higher at 72 h of treatment than that of CH-Pg-NL. Our previous study reported that P-CH-Pg-NL exhibited higher Pg retention in in vitro serum solution (10% FBS solution) than that of CH-Pg-NL and uncoated Pg-NL. The Pg release ratio of P-CH-Pg-NL was only 2.69, while the Pg release ratio of CH-Pg-NL and Pg-NL were 3.43 and 5.68, respectively [8]. However, we selected the 48 h of treatment because of the similar viability of cells in both coated-nanoliposomes. In addition, at 48 h of treatment, the number of viable cells in all treatment groups were comparatively higher than that in 72 h of treatment groups.

### 3.5. Effect of Free-Pg and Pg-Loaded Liposomal Systems against PA-Induced Intracellular ROS Generation

**Figure 4:**
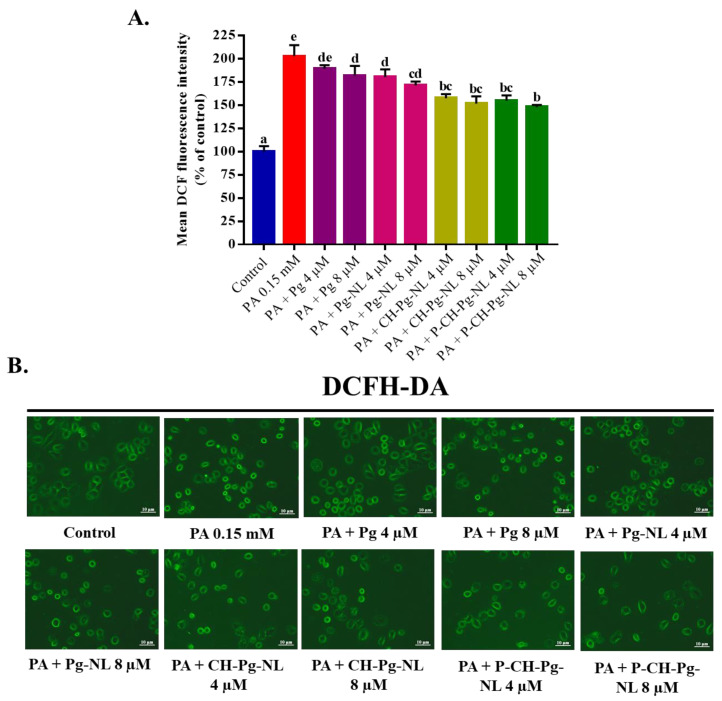
Effects of free Pg and Pg-loaded liposomal systems on PA-induced intracellular ROS production in L02 cells. (**A**) Quantitative determination of ROS as “% of mean DCF fluorescence intensity compared to control”. (**B**) Qualitative determination of ROS by “photographs of DCFH-DA fluorescence intensity of different samples”. Individual alphabet represents the significant difference (*p* < 0.05) of all treatment groups. Here, PA = palmitic acid; Pg-NL = Pelargonidin-*3-O*-glucoside-loaded uncoated nanoliposomes; CH-Pg-NL = Pg-NL coated by chitosan; P-CH-Pg-NL = CH-Pg-NL coated by pectin.

As we know, ROS is a byproduct of aerobic metabolism process, numerous signaling pathways are involved with ROS production. But excess ROS generation can damage the cellular components such as lipids, proteins, and DNA by acting as powerful oxidizing agent [28]. Accumulated evidence suggested that PA can contribute to lipotoxicity via generating excessive ROS [23,28,31]. Therefore, the amelioration effects of free-Pg and Pg-loaded liposomal samples were investigated against PA-induced ROS generation in L02 cells using DCFH-DA probe (Figure 4A,B). According to mean DCF fluorescence intensity, PA treatment significantly (*p* < 0.05) induced the ROS accumulation in L02 cells (202.83 ± 11.84% compared with control 100%). On the flip side, Pg-encapsulated different nanoliposomes significantly (*p* < 0.05) reduced the ROS production compared with PA-treated group after 48 h of pretreatment, while high concentration of pectin–chitosan coated nanoliposomes (P-CH-Pg-NL) exerted maximum ROS reduction property (147.98 ± 2.28%) (Figure 4A,B). Based on the relative reduction of fluorescence intensities by the different samples, we may also find a similar kind of ROS reduction in case of in vivo study. This is because, during oral delivery, all Pg-loaded nanoliposomal systems will go through gastrointestinal harsh environments, e.g., gastrointestinal pH, physiological temperature (37 °C), different ionic strength, serum, proteins, etc., where free-Pg and Pg-loaded uncoated nanoliposomes could be less stable compared to Pg-loaded coated nanoliposomes [8]. In our study, uncoated nanoliposomes (Pg-NL) exerted less ROS reduction capacity, which could be due to the instability of uncoated nanoliposomes in physiological environment [10]. We also observed the least ROS reduction capacity of naked-Pg samples. This is due to the high susceptibility of anthocyanins to harsh environments, e.g., higher pH and higher temperature induced instability, photo and oxidant induced instability, and serum instability [43]. The above data indicate the effective reduction of PA-induced ROS generation in L02 cells after the efficient delivery of Pg-loaded nanoliposomes to cells as well as controlled-release of Pg in cells.

### 3.6. Effect of Free-Pg and Pg-Loaded Liposomal Systems against PA-Induced Intracellular O_2_^•−^ Generation

**Figure 5:**
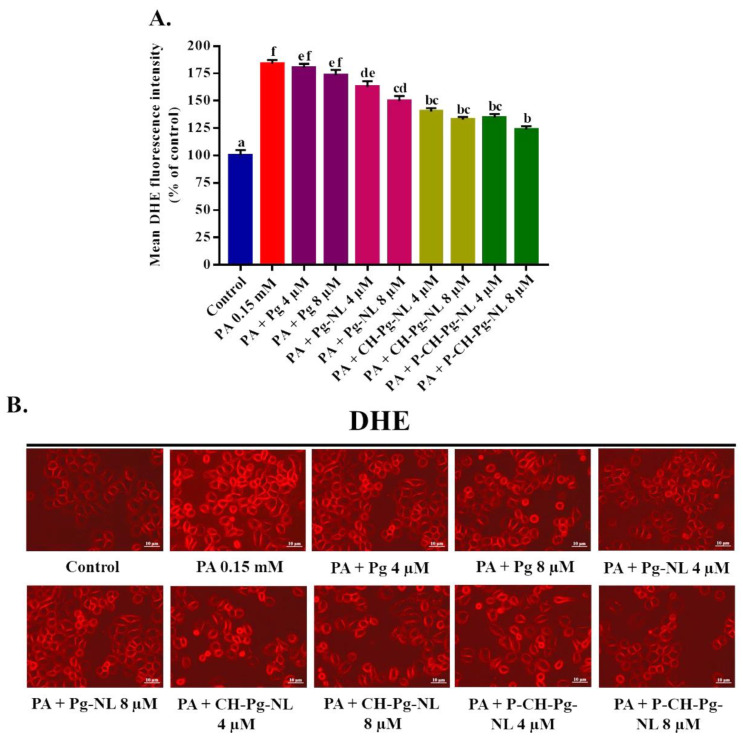
Effects of free-Pg and Pg-loaded liposomal systems on PA-induced intracellular superoxide anion radical (O_2_^•−^) generation in L02 cells. (**A**) Quantitative determination of O_2_^•−^ as “% of mean DHE fluorescence intensity compared to control”. (**B**) Qualitative determination of O_2_^•−^ by “photographs of DHE fluorescence intensity of different samples”. Individual alphabet represents the significant difference (*p* < 0.05) of all treatment groups. Here, PA = palmitic acid; Pg-NL = Pelargonidin-*3-O*-glucoside-loaded uncoated nanoliposomes; CH-Pg-NL = Pg-NL coated by chitosan; P-CH-Pg-NL = CH-Pg-NL coated by pectin.

We further measured the PA-induced intracellular O_2_^•−^ generation in L02 cells using DHE probe. Among all intercellular ROS, superoxide radicals is the primary reactive substance, which can be produced from triplet state of molecular oxygen after the reduction of monovalent [4]. As shown in Figure 5A,B, PA treatment induced the significantly (*p* < 0.05) higher superoxide anion O_2_^•−^ radicals production (183.65 ± 3.66% compared to control 100%). All the liposomal treatments were able to reduce the PA-induced O_2_^•−^ radicals production significantly (*p* < 0.05) compared to PA-treated group. As expected, the coated liposomal samples (CH-Pg-NL and P-CH-Pg-NL) showed the noticeable effect compared to Pg-NL samples, while the naked-Pg samples insignificantly reduced the O_2_^•−^ radicals generation compared to PA-treated group. The above data indicate the effective reduction of PA-induced O_2_^•−^ generation in L02 cells after the efficient delivery of Pg-loaded nanoliposomes to cells as well as controlled-release of Pg in cells.

### 3.7. Effect of Free-Pg and Pg-Loaded Liposomal Systems against PA-Induced Mitochondrial Dysfunction

**Figure 6:**
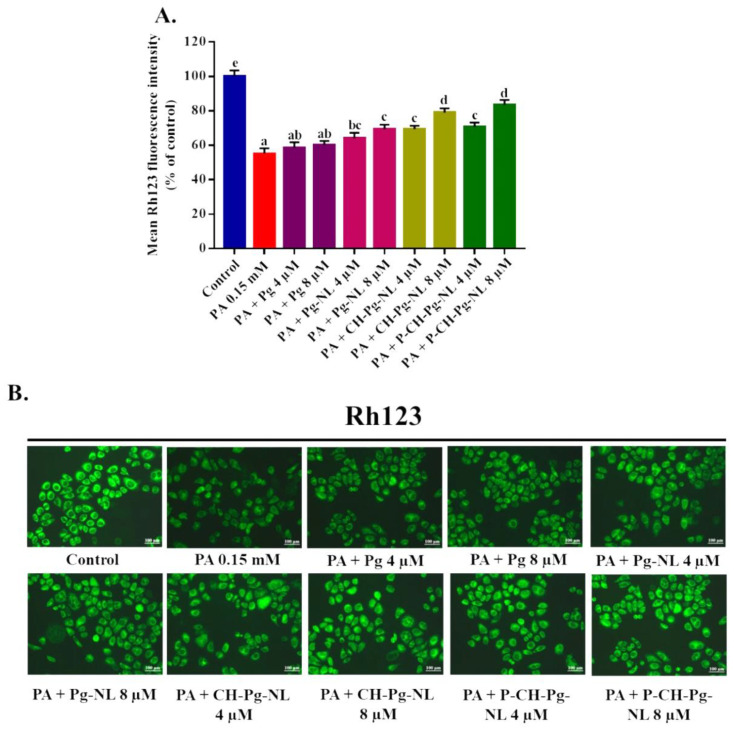
Effects of free-Pg and Pg-loaded liposomal systems on PA-induced mitochondrial membrane potential (MMP) dysfunction in L02 cells. (**A**) Quantitative determination of MMP as “% of mean Rh123 fluorescence intensity compared to control”. (**B**) Qualitative determination of MMP by “photographs of Rh123 fluorescence intensity of different samples”. Individual alphabet represents the significant difference (*p* < 0.05) of all treatment groups. Here, PA = palmitic acid; Pg-NL = Pelargonidin-*3-O*-glucoside-loaded uncoated nanoliposomes; CH-Pg-NL = Pg-NL coated by chitosan; P-CH-Pg-NL = CH-Pg-NL coated by pectin.

A myriad of studies confirmed that mitochondrion plays an important role in ROS homeostasis and its dysfunction. Mitochondrial dysfunctions, such as collapse of mitochondrial membrane potential (MMP), reduction of mitochondrial mass, and others, are the responsible feature of cell death [4,44]. A recent study reported that PA treatment induced the cells death of H9C2 and primary cardiomyoblast of rats via mitochondrial dysfunction [45]. Thus, we detected the mitochondrial membrane potential (MMP) using RH123 fluorescence probe. According to our study, the MMP was excessively declined in PA-treated L02 cells (55.04 ± 3.19%) compared to control cells (100 ± 3.44%). On the contrary, Pg-loaded liposomal systems significantly (*p* < 0.05) restored the MMP compared to PA-treated group, while both CH-Pg-NL and P-CH-Pg-NL samples exhibited similar results (Figure 6A,B). We also found improved results for uncoated nanoliposomes samples compared to free-Pg samples (Figure 6A,B), which indicate the instability of free-Pg samples during treatment in culture medium. Therefore, liposomal samples could be an effective carrier for cellular delivery of bioactive molecules to suppress the mitochondrial dysfunction [46].

### 3.8. Effect of Free-Pg and Pg-Loaded Liposomal Systems against PA-Induced Cellular Glutathione (GSH) Depletion

**Figure 7:**
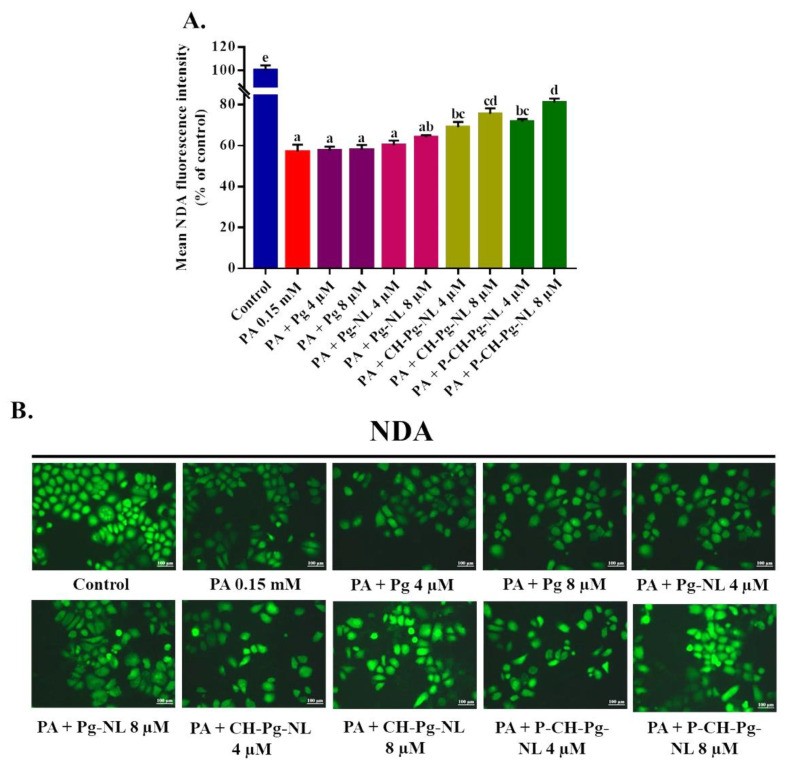
Effects of free-Pg and Pg-loaded liposomal systems on PA-induced cellular GSH depletion in L02 cells. (**A**) Quantitative determination of GSH as “% of mean NDA fluorescence intensity compared to control”. (**B**) Qualitative determination of GSH by “photographs of NDA fluorescence intensity of different samples”. Individual alphabet represents the significant difference (*p* < 0.05) of all treatment groups. Here, PA = palmitic acid; Pg-NL = Pelargonidin-*3-O*-glucoside-loaded uncoated nanoliposomes; CH-Pg-NL = Pg-NL coated by chitosan; P-CH-Pg-NL = CH-Pg-NL coated by pectin.

Glutathione (GSH) is a linear tripeptide, which is composed of three amino acids such as L-glutamine, L-cysteine, and glycine. GSH plays a major role to maintain the redox homeostasis of cells. GSH is capable of preventing cellular components damage caused by ROS [47,48]. Several studies revealed that PA exposure is responsible for cellular GSH depletion [49,50,51]. Therefore, we examined the cellular GSH depletion using NDA fluorescence probe. According to the result, PA exerted the greater GSH depletion with 56.97 ± 3.48% mean NDA fluorescence intensity compared to control (100 ± 4.50%). On the contrary, pretreatment of CH-Pg-NL and P-CH-Pg-NL samples for 48 h significantly restored the cellular GSH content compared to PA-treated group (Figure 7A,B). Even at low concentration, coated nanoliposomes were capable of improving the GSH depletion. Interestingly, Pg-NL samples insignificantly restored the cellular GSH content, while free-Pg samples were unable to ameliorate the GSH depletion. The lower result of uncoated nanoliposomes (Pg-NL) was associated with instability in physiological environment [10], and the lowest result of free-Pg was associated with instability in harsh environments [43].

## 4. Conclusions

Pelargonidin-*3-O*-glucoside (Pg) is a familiar anthocyanin, which processes a wide range of biological activity, including antioxidant, anti-inflammatory, anti-hyperglycemic, antiobesity, antidiabetic, neuroprotective, and anti-cancer activity. Several physicochemical or physiological instabilities, e.g., physiologic pH, temperature (37 °C), oxygen, salt, enzymes, and others limit the therapeutic effect of Pg, though Pg has potential biological activity. Therefore, this study, firstly, encapsulated the pelargonidin-*3-O*-glucoside (Pg) into a pectin–chitosan coated nanoliposomes. Thereafter, this study evaluated the protective effect of dual-coated Pg-loaded nanoliposomal particles against PA-induced nonalcoholic hepatic injury using L02 cells. The DLS, TEM, and HPLC were used to characterize the successful encapsulation of Pg into nanoliposomes, as well as dual coating of nanoliposomes (first chitosan and then pectin coating). The biocompatibility and non-toxic nature of liposomal particles were evaluated using cytotoxicity screening in L02 cells. In addition, the hepatocellular delivery of Pg was further confirmed via cellular uptake study of rhodamine 123-loaded nanoliposomes. Afterward, we confirmed that “pectin–chitosan coated Pg-loaded nanoliposomes” exhibited potential protective activity against PA-induced hepatic injury in L02 cells. In PA-induced L02 cells, our nanoliposomes significantly reduced the intracellular ROS and O_2_^•−^ production, decreased the mitochondrial collapse, and improved the GSH content. In our study, the P-CH-Pg-NL particle was the most efficient system compared to other liposomal systems. Finally, from our study, it is clear that pectin–chitosan coated nanoliposomes can be a suitable carrier for the delivery of hydrophilic compounds (e.g., Pg) with the controlled release property and greater biological activity. However, further in vivo pharmacokinetic studies are needed to understand the controlled release property of pectin–chitosan coated pelargonidin-*3*-*O*-glucoside-loaded nanoliposomes. In addition, in vivo pharmacological studies are required to confirm the improved biological activity of pectin–chitosan coated pelargonidin-*3*-*O*-glucoside-loaded nanoliposomes against palmitic acid-induced nonalcoholic hepatic injury. 

## Figures and Tables

**Table 1:** Characterization of Pg-loaded different nanoliposomal systems using the DLS and HPLC analysis.

		DLS Analysis		HPLC Analysis
Sample	Particle Size (nm)	Zeta Potential (mV)	Polydispersity Index (PDI)	Encapsulation Efficiency (% of EE)
Pg-NL	64.80 ± 0.36	−30.37 ± 0.33	0.21 ± 0.01	28.54 ± 1.36
CH-Pg-NL	252.63 ± 1.92	21.93 ± 0.56	0.13 ± 0.00	53.04 ± 1.19
P-CH-Pg-NL	352.00 ± 4.03	−20.00 ± 0.78	0.24 ± 0.02	61.17 ± 0.90

Here, Pg-NL = Pelargonidin-*3-O*-glucoside-loaded uncoated nanoliposomes; CH-Pg-NL = Pg-NL coated by chitosan; P-CH-Pg-NL = CH-Pg-NL coated by pectin.

## Data Availability

Data is contained within the article.
